# Docetaxel as Salvage Therapy in Highly Pretreated and Drug Resistant Gastrointestinal Carcinomas

**DOI:** 10.4137/cmo.s919

**Published:** 2008-12-03

**Authors:** Martin F. Sprinzl, Sonia M. Wytopil, Anja Dahmen, Stephan Kanzler, Peter R. Galle, Markus Moehler

**Affiliations:** 1 1st Medical Department, Johannes Gutenberg University, Mainz, Germany; 2 Department of Radiology, Johannes Gutenberg University, Mainz, Germany

**Keywords:** chemotherapy, docetaxel, gastric, metastatic, salvage

## Abstract

**Introduction:**

Despite many efforts to develop new chemotherapies for metastatic upper gastrointestinal (GI) cancer, overall prognosis continues to be fatal, particularly in gastric and pancreatic cancer. Many of these patients deserve second-or third-line treatment after failure of first-line chemotherapy. Therefore, we analysed toxicity and response rate of weekly docetaxel after failed upfront regimes in these upper GI cancer patients.

**Patients and Methods:**

Between 2001 and 2006, 18 patients received docetaxel based regimes (35 mg/m^2^ weekly) after informed consent. Response rates were determined using RECIST criteria or tumor progression if clinically evident. Toxicities were graded based on NCI CTC criteria (version 2). Most patients had gastric cancer (13/18). The remaining entities comprised of bilio-pancreatic cancer (5/18).

**Results:**

Docetaxel was administered as 2nd line therapy in 28% (5/18), 3rd line therapy in 56% (10/18) and 4th or 5th line therapy in 17% (3/18). The average docetaxel dose was 38 mg/m^2^ (Median: 35 mg/m^2^) once weekly. Over a treatment duration of 14.7 weeks, the average dosage was 58 gr per patient and week. Overall, docetaxel was well tolerated with only few chemotherapy-associated toxicities (Grade 3/4), including nausea (17%), polyneuropathy (17%), anorexia (11%), neutropenia (6%) and leukopenia (17%).

Docetaxel administration did not achieve any complete responses (CR) and one (5.6%) partial response (PR) was seen (1/18). In addition 5 patients (27.8%) had stable disease (SD), thus inducing a tumor control rate of 33.3% (6/18). Median progression-free survival was 2.4 months for all patients, 2.1 months in the gastric-cancer and 2.4 months in the bilio-pancreatic cancer subgroups respectively. After first docetaxel administration median survival for all patients was 4.5 months, patients with gastric cancer survived for 4.9 months whereas patients suffering from bilio-pancreatic carcinoma survived for 4.2 months. However, taken together 27% (5/18) had a remarkable overall survival of more than 2.5 years.

**Discussion:**

In severely pretreated patients, with documented chemoresistant GI tumors, weekly docetaxel was well tolerated, presented good tumor control rate and overall survival. Therefore, this regimen may be used as salvage treatment in individual patients with upper GI cancers.

## Introduction

Both, patients with metastatic gastric or pancreatic cancers very often present with very short and fatal outcome despite their initial benefits of upfront first-line palliative chemotherapy. These both cancer types contribute to at least ten% of the cancer related mortality in the United States (Jemal et al. 2005; Bray et al. 2002).

Despite many efforts to control tumor progression with new chemotherapeutic agents, the overall mortality has not significantly changed over the last years. (Hohenberger, Gretschel, 2003; Pyrhonen et al. 1995; Murad et al. 1993; Glimelius et al. 1997; Assersohn et al. 2004). For many decades, 5-fluorouracil (5-FU), oral fluorpyrimidines combined with cisplatin have been the basis of most chemotherapy regimens for both cancer types (Waters et al. 1999; Schulze-Bergkamen et al. 2002; Vanhoefer et al. 2000). For pancreatic cancer, gemcitabine has also been established as a palliative standard treatment (Burris et al. 1997), with a median 12 month survival of 18%. (Burris et al. 1997; Rothenberg et al. 1996). Despite many efforts to improve outcome by combinations of these or other cytotoxic substances, the results remain still disappointing with a 5 year survival rate less than 5% after diagnosis of unresectable/metastatic gastric or pancreatic cancer (Louvet et al. 2005; Colucci et al. 2002; Berlin et al. 2002; Jemal et al. 2005; Moehler et al. 2003; Moehler et al. 2005; Wagner et al. 2007; Eickhoff et al. 2006).

Thus, approximately half of all patients facing failure of upfront first line chemotherapy are still in good performance status are willing to and clearly require additional treatment options as second-line treatment protocols.

Herein, taxanes have shown to represent an alternative cytotoxic class with clear antiproliferative activities in gastric and bilio-pancreatic malignancies in vitro and in vivo (Hanauske et al. 1992; Tanaka et al. 1996). Both taxanes, docetaxel or paclitaxel promote microtubule stabilisation by increasing tubulin polymerisation, causing cell cycle arrest in the G2/M phase. After promosing phase II trials have confirmed the activity of docetaxel as single agent, treatment for gastric cancer achieved response rates between 16 and 24% (Bang et al. 2002; Einzig et al. 1996; Mai et al. 1999; Mavroudis et al. 2000; Van Cutsem et al. 2008). Furthermore, the combination of docetaxel, cisplatin and 5-FU has recently been approved as one new standard regimen in advanced gastric cancer (Van Cutsem et al. 2006; Park et al. 2006; Ajani et al. 2005).

To establish a possible salvage therapy in gastric and pancreatic cancer after failure of standard upfront therapies, we analysed the tolerability and clinical effects of a weekly docetaxel regimen in pretreated and chemo-resistant patients with gastric or pancreatic cancers.

## Patients and Methods

### Patients

After informed consent, patients were consecutively recruited into the monocentric study. Eligible patients had pretreated histological proven gastric or bilio-pancreatic adenocarcinomas. All patients had measurable lesions as defined by RECIST criteria or quantifiable non target lesions. Patients who had no previous chemotherapeutic treatment were excluded.

### Administration of study drugs and dose adjustment

Assigned patients received docetaxel as weekly intravenous infusion over 60 minutes. After three subsequent weekly administrations docetaxel was omitted for one week. The mean docetaxel dose reached 38 mg/m^2^ body surface. Antiemetic pre-medication consisted of granisetron (1 mg) and dexamethasone (8 mg). If indicated, prophylactic anti-anaphylactic and anti-emetic treatment included dexamethason 8 mg throughout the first two days after chemotherapy once daily. Dose adjustments of docetaxel were performed if side effects associated to chemotherapy were graded as severe or life threatening. Patients received the palliative treatment of docetaxel between July 2001 and May 2006 (57 months). All side effects were defined following the National Cancer Institute common toxicity criteria (NCI-CTC), version 2 (Moehler et al. 2005).

After first occurrence of chemotherapy associated side effects chemotherapy was stopped until associated toxicity disappeared. Recurrent side effects lead to 20% dose reductions of docetaxel for up to two times. Docetaxel was administered until disease progression or unacceptable and persisting toxicity or withdrawal of consent.

### Study evaluations

At baseline up to five measurable lesions per organ and ten lesions in total were to be identified as target lesions. These were measured using computed tomography (CT), and recorded according to the RECIST system (Response Evaluation Criteria In Solid Tumors) (Therasse et al. 2000). The sum of the longest diameters for all target lesions was used as a reference for determining objective tumor response. Quantifiable non target lesions were chosen alternatively to monitor treatment response if required. Tumor responses were evaluated after an average of 11 weeks representing about three cycles of chemotherapy. Patients were followed for an average of 6.9 months (median 4.5 months). Complete response (CR) was defined as the disappearance of all target and non-target lesions with no new lesions. Partial remission (PR) was defined as a reduction of 30% or more in the sums of the longest diameters of all measurable lesions relative to baseline with no new lesions; Stable disease (SD) was defined as neither sufficient shrinkage to qualify for PR nor sufficient increase to qualify for progressive disease (PD) with no new lesions; PD was defined as ≥20% increase in the sum of the longest diameters or the occurrence of non-target lesions (e.g. pleural effusion or ascites). Safety and tolerability were assessed weekly by regular clinical examinations and assessments of adverse events during administration of chemotherapy. Monitored parameters of safety and tolerability referred to disease symptoms and haematological and biochemical parameters.

## Results

### Patient characteristics

Eighteen patients were evaluated in this study. The median age of all patients was 59.5 years. In 72% (13/18) of all patients the primary tumor site was gastric and in 28% (5/18) bilio-pancreatic, with only one patient with cholangiocarcinoma. All patients had metastatic disease and in 78% (14/18) two or more organs were involved (Table 1). One third showed ascites or radiological signs suspicious for peritoneal carcinomatosis.

Every patient with gastric cancer had received a 5-FU or capecitabine based regimen prior to docetaxel. These pretreatments were combined with cisplatin, irinotecan or etoposide, in 84% (11/13), 69% (9/13) or in 23% (3/13), respectively. Thus in the gastric cancer subgroup all patients had previously been treated with either cisplatin or irinotecan. Two patients additionally had radio-chemotherapy pretreatment. Also patients with bilio-pancreatic cancer were previously treated with gemcitabine based chemotherapy schedules, which was administered as first line in 80% (4/5). Patients with bilio-pancreatic carcinoma also had received 5-FU or oxaliplatin in 60% (3/5) each (Table 2).

#### Safety

A median number of nine chemotherapy cycles was administered during the observed study period. Dose administration delays became mandatory in 61% (11/18) and the absolute number of treatment delays accounted for 16% of all docetaxel infusions. Dose reductions were performed in 17% (3/18) of all patients and were limited to only one dose reduction of 20%. The main reason for discontinuing study treatment was disease progression. The primary reason responsible for treatment adjustments (dose reduction and treatment delay), were leucopenia and infection. In contrast, the most common chemotherapy-associated side effects were anorexia, edema, nausea/vomiting and toxic polyneuropathy. The incidence of haematological toxicities, grade 3 to 4, with an overall rate of 22%, (4/18) was low compared to other docetaxel infusions (Van Cutsem et al. 2006) (Tables 3a/b). Fatal infectious complications (NCI-CTC, Grade 4) were observed in one case but could not be attributed directly to chemotherapy.

#### Response rates

The tumor control rate (CR + PR + SD) of all patients for the entire treatment period including all assessments prior to discontinuation was 33% (6/18). The mainstay of patients showed stable disease whereas only one patient showed a partial response and no patient developed complete responses. Analysis of the patients suffering from gastric cancer revealed a tumor control rate of 38.5% (5/13). Bilio-pancreatic tumors showed a tumor control rate of only 20% (1/5) (Table 4).

#### Progression-free survival, treatment failure and overall survival

The median follow-up was 57 months at the last data cut-off. At this time, 15 of 18 patients had died. Disease progression was responsible for mortality in 87% of all cases during this study. One patient died from sepsis-related complications, which were not prompted by chemotherapy associated neutropenia. Median progression-free survival, median time to treatment failure and median overall survival are listed in Table 5.

## Discussion

Although chemotherapy regimens for gastric cancer and bilio-pancreatic cancer offer improvements in survival or quality of life, nearly all patients eventually progresses. The endpoints chosen in this clinical analysis was time till progression after initiation of docetaxel and overall survival after first administration of docetaxel. In this analysis we observed an overall survival of 6.9 months (median 4.5 months) and a time till progression of 3.1 months (median 2.4 months) after initiation of docetaxel. As many patients have received multiple pre-treatments with standard chemotherapy protocols, response rates were predictably low. In this highly pretreated patient population overall response rates of 33.3% (PR and SD) are still remarkable as cross resistance has been described for multiple malignant entities.

When patient histories, disease status and other factors were examined for their effects on clinical outcome, those patients who were in better general health (good performance status, low tumor burden) were more likely to achieve a response and less likely to have a progression event or die, regardless of the treatment chosen for palliative chemotherapy. Results from prospective clinical trials investigating the effects of peritoneal carcinomatosis ascites on patient survival predicted a mean survival for gastric cancer of 3.1 months. For pancreatic cancer, the results were even more fatal showing a mean survival of 2.1 months if peritoneal effusions have developed (Chu et al. 1989; Rau et al. 1996; Sadeghi et al. 2000). Thus, our limited response rates to docetaxel may be partially explained by the special characteristics of our study population, as our mostly highly pretreated patients developed extended tumor stages before salvage chemotherapy could be initiated and ascites and radiological indicators for peritoneal carcinomatosis were present in 33%, also in agreement to other trials (Sadeghi et al. 2000).

Despite fatal prognoses some individual patients showed an overall survival, which was longer than expected. This indicates that some malignancies with specific biological patterns might respond to salvage therapy. Whereas the median survival was limited, remarkably enough some patients survived more than 5 years, with the longest overall survival of 8.4 years for a patient suffering from metastatic gastric carcinoma. Another patient diagnosed with cholangiocarcinoma and an overall survival of more than two years indicating that some patients might benefit from salvage treatment. Taken together about 27% (5/18) of all individuals were able to experience an overall survival of more than 2.5 years. After all, the primary tumor site had a substantial impact on prognosis. In this context it was observed that pancreatic neoplasm were associated with worse outcomes than gastric carcinoma.

Given that not all patients will benefit from salvage treatment, safety of chemotherapy is an essential issue, which was addressed in this retrospective analysis. We conducted an analysis of all patient out-clinic visits and were able to extract all relevant side effects. Despite extended tumor disease only one patient experienced a life threatening complication, which was not conclusively attributed to docetaxel. Most significant side effects (NCI-CTC, grade 3–4) were caused by haematological toxicity, affecting 22% of all patients. In this observation polyneuropathy and nausea, both in 16.5% of all patients, were other common toxicities (NCI-CTC, grade 3–4) of docetaxel. In contrast, clinical trials investigating single agent docetaxel as fist-line treatment have shown a much higher leucopenia and/or neutropenia rates of 80 to 90% (Bang et al. 2002; Einzig et al. 1996; Sulkes et al. 1994; Lorenzen, 2007; Van Cutsem et al. 2006). These results are also in agreement with trials investigating docetaxel as second-line chemotherapy, which report a similar haematological toxicity affecting 14% to 18% of all patients (Graziano et al. 2000; Giuliani et al. 2003). Therefore docetaxel is a manageable salvage treatment option with potential benefit for patients with refractory gastric and bilio-pancreatic carcinoma and further prospective trials are warranted to confirm this retrospective analysis.

## Figures and Tables

**Table 1:** Patient characteristics.

	Primary tumor site	
	Gastric (n = 13)	Bilio-pancreatic (n = 5)
Gender (male/female)	13/0	1/5
Age (median), years (range)	58 (47–69)	63 (56–68)
Metastatic sites		
Hepatic	10	5
Pulmonal	4	0
Lymphatic/nodal	10	4
Skeletal	2	–
Peritoneal	1	–
other sites	3	–
No. of metastatic sites		
0	–	–
1	3	1
2	4	4
≥3	6	0

**Table 2:** Pre-treatments of study population.

	Primary tumor site	
	Gastric (%)	Bilio-pancreatic (%)
5-FU/Capecitabine	13 (100)	4 (80)
Cisplatin	11(85)	–
Irinotecan	9 (69)	–
Etoposid	3 (23)	–
Interferon	1(7.7)	–
Gemcitabine	–	5 (100)
Oxaliplatin	–	3 (60)

**Table 3a:** Haematological toxicities.

	No. Patients (%)					
NCI-CTC Grade	1	2	3	4	3–4	1–4
Leukopenia	–	1	2	1	3 (16.5)	4 (22)
Neutropenia	–	1	1	–	1(5.5)	2 (11)
Anemia	3	2	–	–	–	5 (28)

**Table 3b:** Non–haematological toxicities.

	No. Patients (%)					
NCI-CTC Grade	1	2	3	4	3–4	1–4
Nausea/vomiting	8	5	3	–	3 (16.5)	6 (33)
Polyneuropathy	2	1	3	–	3 (16.5)	6 (33)
Anorexia	4	4	2	–	2 (11.0)	10 (56)
Dyspnea	1	2	1	–	1 (5.5)	4 (22)
Edema	2	5	1	–	1 (5.5)	8 (44)
Fatigue	4	2	1	–	1 (5.5)	7 (39)
Mucositis	2	2	1	–	1 (5.5)	5 (28)
Alopecia	2	2	–	–	–	4 (22)
Diarrhoea	2	4	–	–	–	6 (33)

**Table 4:** Response.

	Primary tumor site		
	Gastric (%)	Bilio-pancreatic (%)	Total (%)
Complete Response	0 (0)	0 (0)	0 (0)
Partial response	1 (7.7)	0 (0)	1 (5.5)
Stable disease	4 (30.8)	1 (20)	5 (27.7)
Tumor control rate (CR + PR + SD)	5 (38.5)	1 (20)	6 (33.3)
Progressive disease	8 (61.5)	4 (80)	12 (66.7)

**Table 5:** Survival.

	Primary tumor site		
	Gastric months (Median)	Bilio-pancreatic months (Median)	Total months (Median)
Progression free survival	2.9 (2.1)	3.5 (2.4)	3.1 (2.4)
Survival after docetaxel	6.5 (4.9)	9.5 (4.2)	6.9 (4.5)
Overall survival	25.9 (22.8)	22.1 (20.6)	24.3 (20.6)
